# Ecotypic Variation in Photosynthesis, Stomatal Conductance, and Water Use Efficiency of *Illicium lanceolatum* in Response to Light Intensity Under Drought and Recovery

**DOI:** 10.3390/plants15030407

**Published:** 2026-01-29

**Authors:** Yonghui Cao, Benzhi Zhou

**Affiliations:** 1 Research Institute of Subtropical Forestry, Chinese Academy of Forestry, Hangzhou 311400, China; benzhi_zhou@126.com; 2 Qianjiangyuan Forest Ecosystem Research Station, National Forestry and Grassland Administration, Hangzhou 311400, China

**Keywords:** ecotype selection, *Illicium lanceolatum*, medicinal plant, photosynthetic parameters, stomatal conductance, leaf-water use efficiency, water stress, recovering, light intensity

## Abstract

Increasingly frequent extreme droughts threaten forest vegetation and highlight the need to identify drought-tolerant germplasm. To support conservation and cultivation of *Illicium lanceolatum*, we investigated ecotypic differences in photosynthetic responses to short-term drought and rewatering under varying light intensity. One-year-old seedlings from four *I. lanceolatum* ecotypes originating from the Zhejiang (Lin’an, LA; Kaihua, KH), Jiangxi (Wu’ning, WN), and Fujian (Nan’ping, NP) provinces in China were subjected to drought stress by withholding irrigation and subsequent rewatering. Photosynthesis–light response curves were measured before drought; 2, 4, and 7 days after the last watering; and following rewatering. Short-term drought significantly affected photosynthetic traits in an ecotype-dependent manner. Maximum net photosynthetic rate, light saturation point, light compensation point, and apparent quantum yield increased during drought, indicating enhanced utilization of both high and low light. After rewatering, stomatal conductance increased significantly in the WN and KH ecotypes but declined in the NP ecotype when compared with those under the initial water supply. Instantaneous water use efficiency (A/E) recovered rapidly in all ecotypes and exceeded pre-drought levels. Under light intensity above 1500 µmol·m^−2^·s^−1^, stomatal conductance exhibited a significant nonlinear relationship with water use efficiency. Overall, these physiological responses indicate that *I. lanceolatum* is moderately drought-tolerant and exhibits mild sensitivity to soil water variation. The WN and KH ecotypes showed superior improvement in water use efficiency under drought and high light, suggesting their potential for breeding drought-resistant cultivars and for afforestation in drought-prone environments.

## 1. Introduction

The increasing frequency and intensity of drought events driven by global climate change pose serious threats to plant diversity, ecosystem productivity, and species distribution worldwide [1,2,3,4]. Adjusting their physiological traits to cope with water scarcity is, therefore, essential for elucidating mechanisms of drought adaptation and resilience [5,6,7]. Among these traits, stomatal regulation plays a central role in balancing carbon gain and water loss. As the gatekeepers of leaf gas exchange, stomata strongly influence plant performance under conditions of elevated temperature and drought stress [8,9]. In general, stomata respond to declining soil water availability and increasing atmospheric vapor pressure deficit by closing [10,11], which reduces stomatal conductance (*gₛ*) and limits carbon assimilation [9,12,13]. Under drought conditions associated with approximately 30% reduction in annual precipitation, tree net CO_2_ assimilation decreased by 49%, whereas stomatal conductance, transpiration rate, and water use efficiency remained largely unchanged [14]. This indicates that plant species exhibit distinct and dynamic physiological responses to drought. While many studies in wheat report reductions in *g_s_* under heat or drought stress [15,16], other studies show that *g_s_* responses can vary markedly depending on environmental conditions, growth stage, and genotype [9]. These contrasting responses suggest that plant water use strategies are highly dynamic and context-dependent [17].

Water use efficiency (WUE) is a key indicator of plant drought adaptation [11]. Instantaneous water use efficiency (*iWUE*), defined as the ratio of carbon assimilation to transpiration, reflects how effectively plants assimilate carbon under water-limited conditions and is particularly informative during drought [6,13,18,19,20,21]. Improving *iWUE* is, therefore, critical for enhancing plant performance under future climate scenarios [22,23]. Although drought often reduces *g_s_* and *iWUE* [21], moderate drought can increase *iWUE* by constraining water loss more strongly than carbon gain [24], whereas severe drought generally leads to declines in both traits [25,26].

Drought stress directly affects key determinants of plant productivity, including canopy structure, photosynthesis, and assimilate allocation. Consequently, identifying drought-tolerant ecotypes is increasingly important under ongoing climatic variability [27]. While intraspecific variation in drought tolerance is recognized as a major component of plant adaptation to climate change [4,28], such variation remains poorly characterized in many woody species. Widely distributed species often exhibit pronounced ecotypic differentiation in functional traits due to local environmental conditions [29,30], yet comparative studies examining ecotypic variation in *iWUE* and stomatal regulation under drought are still limited.

*Illicium lanceolatum* A.C. Smith, a medicinal plant commonly known as poisonous eight-angle [31], is an endemic evergreen tree species restricted to southern China. It is an important medicinal and aromatic plant, valued for its anti-inflammatory and analgesic properties and as a major natural source of shikimic acid, a key precursor of the antiviral drug oseltamivir (Tamiflu) [32,33]. Due to overharvesting and habitat degradation, *I. lanceolatum* has become an endangered species in China, highlighting the urgent need for conservation, germplasm protection, and large-scale artificial cultivation.

Adaptation to macro- and micro-climatic variation may depend on genetic differentiation among ecotypes [34]. Increasing water scarcity poses a major challenge for cultivated tree species such as *Illicium lanceolatum*. Consequently, identifying genotypes with high leaf *iWUE* [13,35]) is crucial for improving the sustainability of future artificial forests. In order to improve *iWUE* in cultivated plants, Gilbert et al. (2011) proposed either reducing *g_s_* under well-watered conditions or enhancing stomatal responsiveness in the early stages of drought stress [36]. However, how the coupled effects between the drought and the light intensity influence *g_s_* and *iWUE* across different *I*. *lanceolatum* ecotypes remains largely unexplored.

In its natural habitats, *I. lanceolatum* occurs discontinuously in valley broad-leaved forests at elevations of 600 to 1000 m, often growing in shaded, moist, and fertile soils alongside species such as *Phoebe sheareri Phoebe sheareri*, *Michelia platypetala*, and *Manglietia chingii* [37]. Although the species is shade-tolerant and morphologically robust, wild populations have declined sharply due to intensive exploitation. Despite its ecological and economic importance, experimental studies examining the physiological mechanisms underlying its responses to water and light availability—particularly under common garden conditions—remain scarce.

Stomatal regulation is a key mechanism by which plants mitigate drought stress [37,38]. Drought-tolerant genotypes often exhibit lower *g_s_* under well-watered conditions or enhanced stomatal sensitivity during early drought, thereby conserving soil water and maintaining higher *iWUE* [39]. However, how *g_s_* and *iWUE* jointly respond to the combined effects of drought and light intensity across different *I. lanceolatum* ecotypes remains unclear. In this study, we compared the dynamic responses of photosynthetic traits, *g_s_* and *iWUE* among four *I. lanceolatum* ecotypes under varying drought durations and subsequent rewatering. Our objectives were to (1) evaluate the sensitivity of photosynthesis, *g_s_*, and *iWUE* to soil water availability under different light conditions and (2) identify ecotypic differences in drought tolerance. Ultimately, this work aims to select drought-tolerant ecotypes suitable for future cultivation and afforestation under climate change, thereby supporting ex situ conservation and sustainable utilization of *I. lanceolatum* genetic resources.

## 2. Results

### 2.1. Effects of Drought Stress on Photosynthetic Properties in Four I. lanceolatum Ecotypes

**Figure 1:**
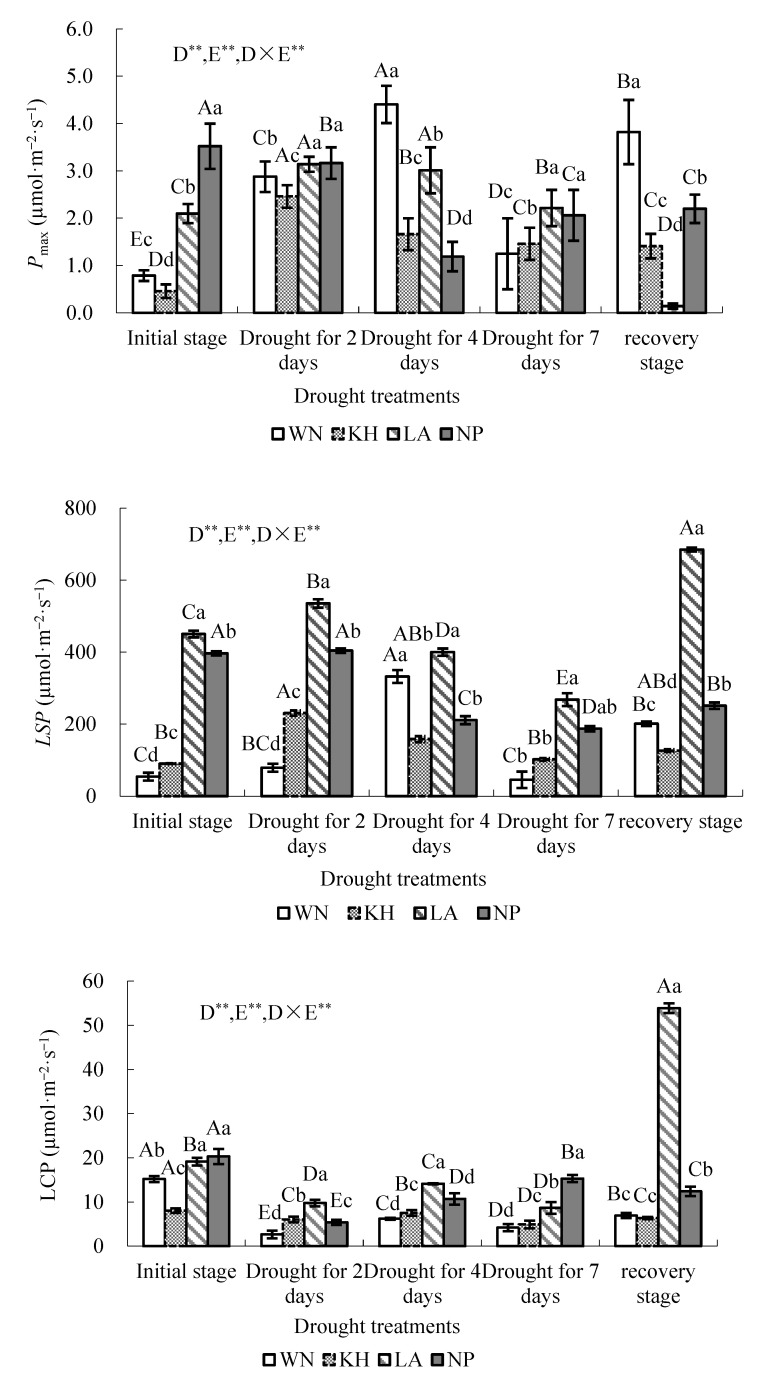

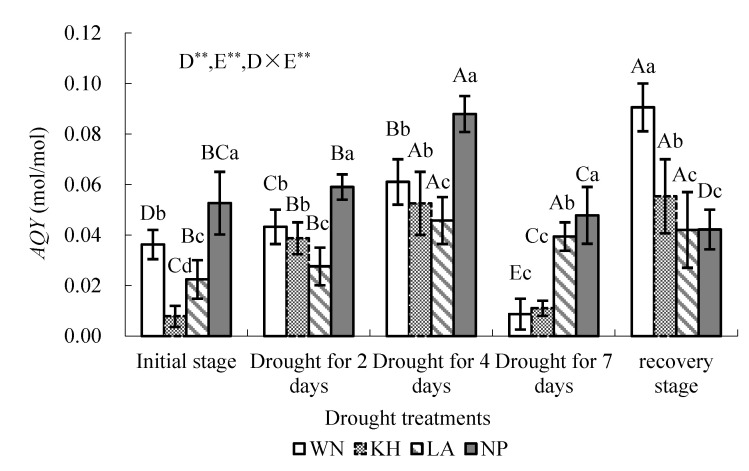
Photosynthesis-light response characteristics of *Illicium lanceolatum* seedlings from different ecotypes under varying drought conditions. Each data point represents the mean ± standard deviation (SD) of more than ten measurements obtained from leaves of 5 individual seedlings (*n* ≥ 10). Different capital letters (A, B, C, D, etc.) listed on each histogram indicate significant differences among drought treatments for the same ecotypes (*p* < 0.05); different lower case letters (a, b, c, d, etc.) listed on each histogram indicate significant differences among the ecotypes under the same drought treatment (*p* < 0.05). *P*_max_: maximum net photosynthetic rate; LSP: light saturation point; LCP: light compensation points; AQY: apparent photon quantum efficiency. ** indicates *p* < 0.01. D represents drought treatment, E represents ecotype, and D × E represents the interaction effect between D and E.

Photosynthetic parameters showed clear ecotypic differences during the short-term drought stress (DS) and subsequent recovery (Figure 1). The maximum net photosynthetic rate (*P*_max_), which reflects the maximum photosynthetic capacity of leaves under optimal conditions [40,41], was significantly influenced by drought treatment, ecotype, and their interaction (two-way variance analysis, *p* < 0.01). Under the initial water stage, after 4 days since the last watering, and after rewatering, *P*_max_ differed significantly among the four ecotypes (*p* < 0.05). After 2 days and 7 days without watering, *P*_maxs_ of the LA and NP ecotypes were significantly higher than those of WN and KH (*p* < 0.05). The temporal dynamics of *P*_max_ varied among ecotypes. In the LA and KH ecotypes, *P*_max_ increased rapidly soon after 2 days of drought and then declined (Figure 1). In contrast, *P*_max_ in the WN ecotype continued to increase and reached the highest level after 4 days of drought. Following rewatering, *P*_max_ continued to decrease in the LA and KH ecotypes, whereas it increased in the WN ecotype.

During the DS treatment, *P*_max_ of the four ecotypes was positively correlated with soil water content (*W*_m_). LSD analysis showed that *P*_max_ of the WN seedlings differed significantly between the DS treatment period and the initial watering stage (*p* < 0.05), with the NP ecotype showing the highest value, followed by LA ecotype. After 2 days of drought, *P*_max_ increased significantly in all ecotypes except NP, and no significant difference was observed between LA and NP, which maintained the highest *P*_max_ level. After 4 days of drought, *P*_max_, again, differed significantly among all ecotypes (*p* < 0.05). These results indicate that ecotypes differ markedly in the magnitude and temporal patterns of *P*_max_ responses to drought, likely reflecting distinct stomatal and physiological adjustment strategies under progressive water limitation.

Light saturation points (LSPs) and light compensation points (LCPs) reflect a plant’s ability to utilize high and low light intensities, respectively [40]. Two-way variance analysis showed that drought treatments, ecotype, and their interaction significantly affected LSPs and LCPs across the four ecotypes *(p* < 0.01) (Figure 1).

Under initial watering, after 2 days of drought, and after rewatering, LSPs differed significantly among the four ecotypes (*p* < 0.05). After 4 days of drought, the LSPs of WN and LA ecotypes were significantly higher than those of KH and NP, while after 7 days, the LA ecotype had the highest LSP (*p* < 0.05). *W*_m_ decreased to 23.81%–33.77% after 2 days of drought (Table 1), during which LSPs increased in all ecotypes. After 4 days, WN seedlings reached the highest LSP (332.30 µmol·m^−2^·s^−1^), whereas the other ecotypes declined as drought progressed. Across all drought stages, the LSP of LA remained consistently higher than the other ecotypes. Rewatering restored LSPs, which exceeded initial values in WN, LA, and KH. LSD analysis indicated significant differences between drought and initial water conditions for specific ecotypes at different time points: LA at all stages, NP after 4 days, WN on day 4, and KH on day 2 (*p* < 0.05).

LCP, the light intensity at which photosynthesis balances respiration [42], decreased during drought and recovered after rewatering [42], with a marked increase observed only in LA. Initially, LCPs of LA and NP were higher than WN and KH (*p* < 0.05). As drought progressed, LCPs differed significantly among ecotypes (*p* < 0.05). After rewatering, LA maintained the highest LCP, while LA and NP were significantly higher than WN and KH.

The apparent photo quantum efficiency (AQY) reflects the efficiency of light utilization under low light [43]. Two-way variance analysis revealed that drought, ecotype, and their interaction significantly affected AQY *(p* < 0.01; Figure 1). AQY increased in all ecotypes until day 4 of drought. LSD analysis showed that AQY of NP was significantly lower than initial values (*p* < 0.05), whereas AQY of LA, WN, and KH increased significantly (*p* < 0.05). Initially, AQY differed significantly among ecotypes (*p* < 0.05). As drought progressed, differences between WN and KH became nonsignificant. After 2 and 4 days, WN and KH had higher AQY than LA but lower than NP (*p* < 0.05). By day 7, WN and KH had significantly lower AQY than LA and NP. After rewatering, AQY in WN and KH remained higher than in LA and NP. These results indicate that LSP, LCP, and AQY exhibit ecotype-specific dynamic responses to drought, reflecting differences in light use strategy and stomatal adaptation among *I. lanceolatum* ecotypes.

### 2.2. Effect of Drought Stress on Stomatal Conductance of I. lanceolatum Ecotypes

**Figure 2:**
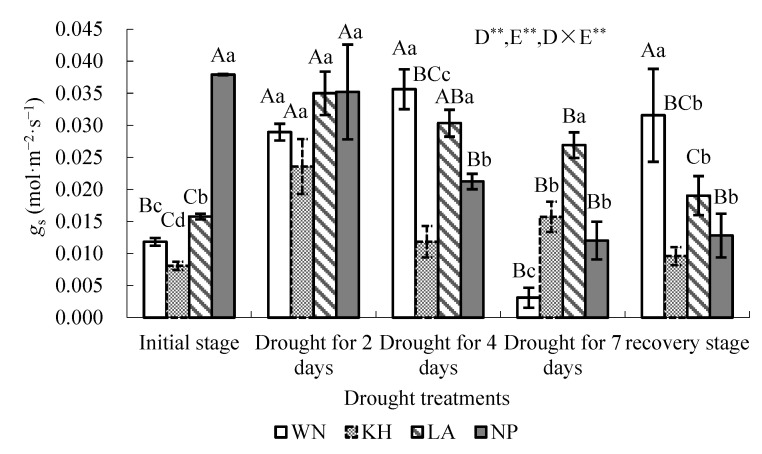
Variation in average *gₛ* of *Illicium lanceolatum* seedlings under drought stress at *PAR* > 1500 µmol·m^−2^·s^−1^. Each point represents the mean ± SD from more than ten measurements on leaves of five individual seedlings (*n* ≥ 10). Capital letters (A, B, C, etc.) listed on each histogram in the figure indicate significant differences among drought treatments for the same ecotypes according to LSD test (*p* < 0.05); and different lower case letters (a, b, c, d, etc.) listed on each histogram indicate significant differences among ecotypes under the same drought treatment according to LSD test (*p* < 0.05). *gₛ*: stomatal conductance, *PAR*: photosynthetic active radiation. ** indicates *p* < 0.01. D represents drought treatment, E represents ecotype, and D × E represents the interaction effect between D and E.

The variation of average *gₛ* under *PAR* > 1500 µmol·m^−2^·s^−1^ for the four ecotypes during drought stress is shown in Figure 2. Two-way variance analysis showed that drought treatments, ecotype, and their interaction significantly affected the *gₛ* across the experimental period *(p* < 0.01). LSD analysis revealed that *gₛ* differed significantly both among water treatments within the same ecotype and among ecotypes under the same water treatment.

**Figure 3:**
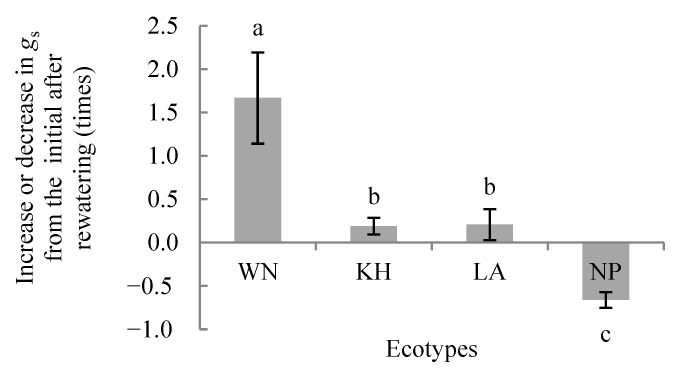
Relative change in *gₛ* from the initial stage after rewatering in seedlings of different *Illicium lanceolatum* ecotypes. Each point represents the mean ± SD from more than ten measurements on leaves of five individual seedlings (*n* ≥ 10). Lower case letters (a, b, c, etc.) above each bar indicate significant differences among ecotypes according to LSD test (*p* < 0.05). *gₛ*: stomatal conductance.

At the initial watering stage, NP exhibited the highest *gₛ*, while KH had the lowest (Figure 2). During drought, *gₛ* trends varied among ecotypes. In NP, *gₛ* decreased continuously with drought and remained below initial levels after rewatering (*p* < 0.05), indicating strong limitation by soil moisture under high light. In contrast, WN *gₛ* increased rapidly during short-term drought (Figure 3), peaking at 4 days when soil water content dropped to 19.64 ± 0.34% (Table 1), then declined sharply by day 7. After rewatering, *gₛ* recovered significantly above initial levels (*p* < 0.05).

For the LA ecotype, *gₛ* increased rapidly during the first 2 days of drought, then declined as drought progressed, and partially recovered after rewatering, with significant differences compared to the initial water stage (*p* < 0.05). In the NP ecotype, *gₛ* decreased rapidly during drought and increased slightly after rewatering, but remained significantly lower than the initial water stage (*p* < 0.05), indicating that soil moisture strongly limits *gₛ* under high light (>1500 µmol·m^−^^2^·s^−^^1^). In KH, *gₛ* initially increased during early drought, then declined sharply, reaching a minimum at 7 days when soil water content dropped to 14.96 ± 0.09% (Table 1), and slightly exceeded initial levels after rewatering. For both LA and KH ecotypes, *gₛ* recovered and showed a slight increase after rewatering compared with the initial stage, although these changes were not statistically significant (*p* > 0.05). Overall, under high light (>1500 µmol·m^−^^2^·s^−^^1^), the ecotypes displayed distinct *gₛ* dynamics in response to soil water limitation. WN and LA showed rapid short-term increases in *gₛ* during early drought, while NP was consistently constrained by low soil moisture, and KH exhibited an intermediate response.

### 2.3. Effect of Drought Stress on Water Use Efficiency of I. lanceolatum Ecotypes

**Figure 4:**
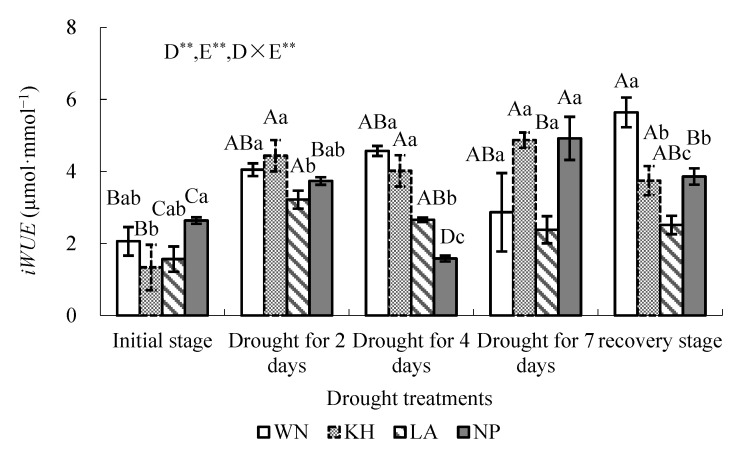
Variation in average *iWUE* of *Illicium lanceolatum* seedlings from different ecotypes under drought stress at *PAR* > 1500 µmol·m^−2^·s^−1^. Each point represents the mean ± SD from more than ten measurements on leaves of five individual seedlings (*n* ≥ 10). Upper case letters above bars indicate significant differences among drought treatments within the same ecotype, and different lower case letters indicate significant differences among ecotypes under the same drought treatment (LSD test, *p* < 0.05). ** indicates *p* < 0.01. D represents drought treatment, E represents ecotype, and D × E represents the interaction effect between D and E.

**Figure 5:**
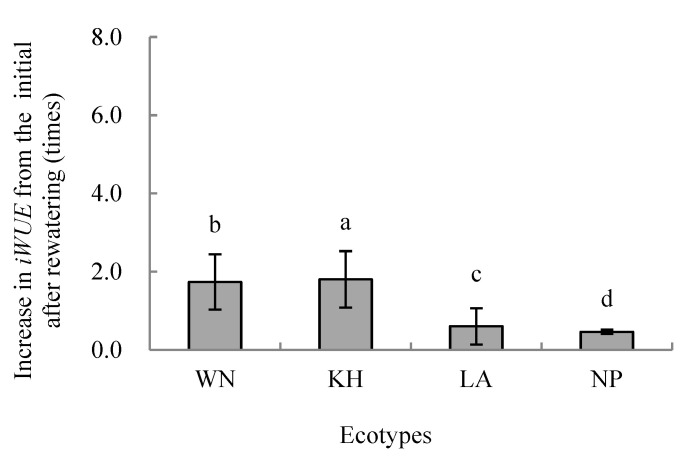
Relative increase in (*iWUE*) from the initial stage after rewatering in seedlings of different *Illicium lanceolatum* ecotypes. Each bar represents the mean ± SD from more than ten measurements on leaves of five individual seedlings (*n* ≥ 10). Lower case letters above bars indicate significant differences among ecotypes according to the LSD test (*p* < 0.05).

Two-way variance analysis showed that drought treatments, ecotype and their interaction significantly affected the *iWUE* of each ecotype *(p* < 0.01) (Figure 4). LSD analysis revealed that at the initial watering stage, NP had the highest *iWUE*, while KH and LA were lower, with a significant difference only between KH and NP (*p* < 0.05) (Figure 4). During short-term drought, *iWUE* increased in all ecotypes except NP on day 4, which was slightly lower than its initial value. By day 7, *iWUE* of KH, LA, and NP had increased significantly compared with initial conditions (*p* < 0.05), whereas WN showed no significant change. After rewatering, *iWUE* of all ecotypes increased significantly above initial values (*p* < 0.05; Figure 4), with WN and KH exhibiting the highest *iWUE* (Figure 5).

Temporal patterns varied among ecotypes. KH showed a prolonged rise in *iWUE* after drought, followed by WN, whose *iWUE* decreased by day 7 but rebounded after rewatering to exceed initial levels (Figure 4). LA consistently displayed lower *iWUE* than WN and KH during short-term drought. These results indicate that under short-term soil drought, higher light conditions promote *iWUE* in all ecotypes. Moreover, the relative increase in *iWUE* from the initial stage after rewatering differed significantly among ecotypes (Figure 5), highlighting ecotype-specific adaptation strategies in carbon assimilation and water use under drought and recovery.

### 2.4. Relationship Between Stomatal Conductance and Water Use Efficiency

**Figure 6:**
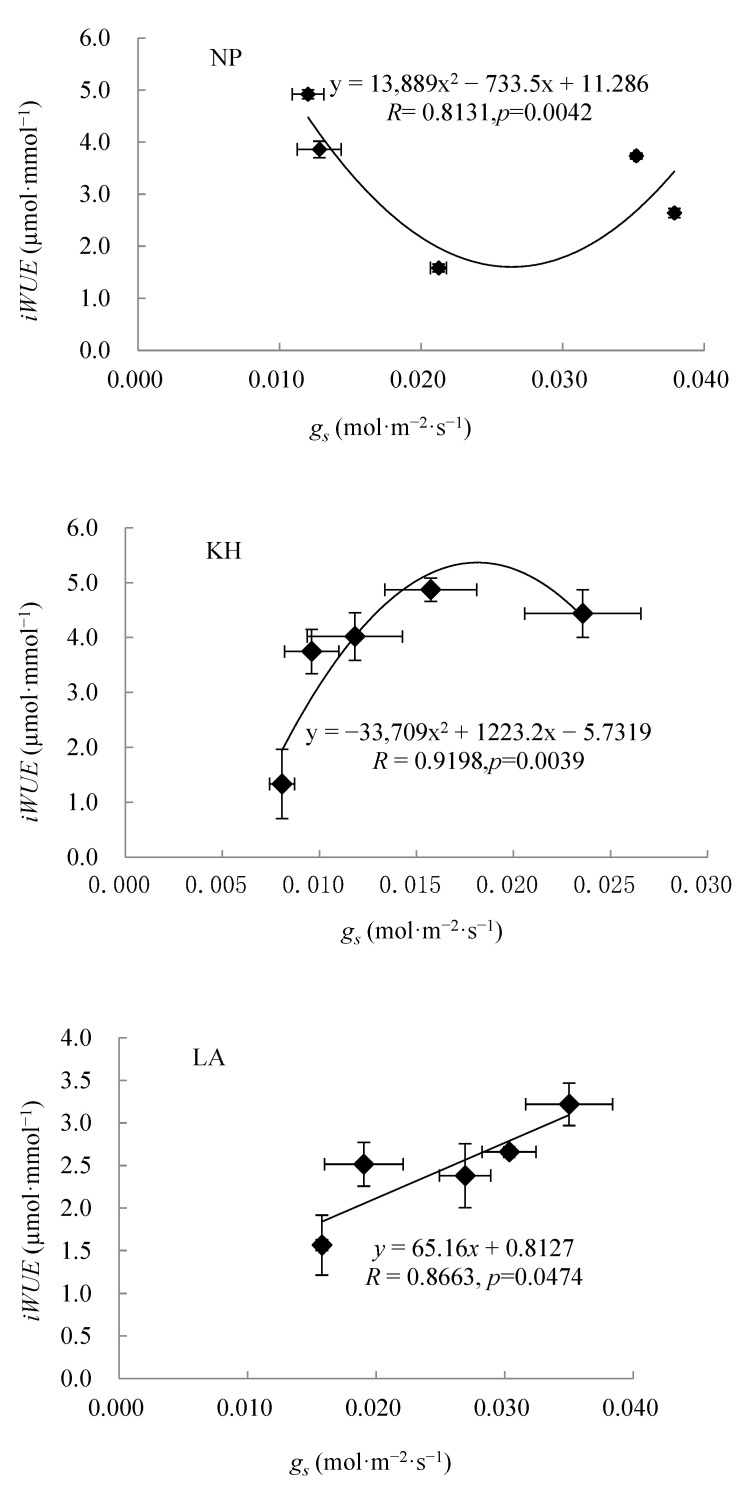

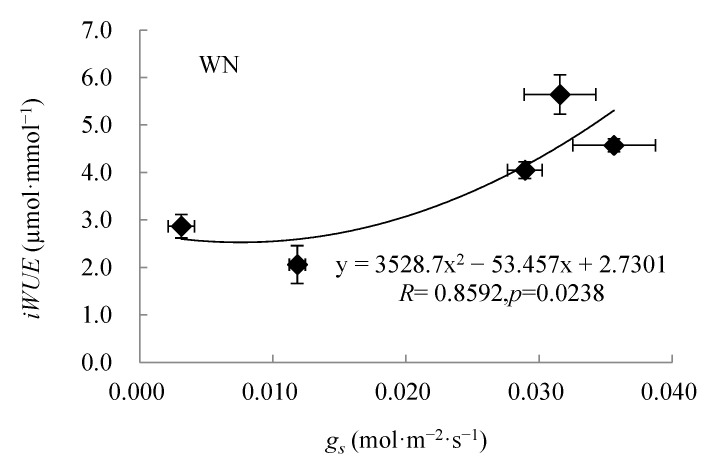
Relationship between means of *gₛ* and *iWUE* measured under *PAR* > 1500 µmol·m^−2^·s^−1^ in seedlings of four ecotypes of *Illicium lanceolatum* under drought treatments. NP—Nan’ping ecotype; KH—Kai’hua ecotype; LA—Lin’an ecotype; WN—Wu’ning ecotype.

Under *PAR* exceeding 1500 µmol·m^−2^·s^−1^, a significant polynomial relationship was observed between mean stomatal conductance *gₛ* and *iWUE* in seedlings of the NP, KH, and WN ecotypes of *I. lanceolatum* across different water treatments. The correlation coefficients (R) ranged from 0.8131 to 0.9198, indicating a strong association between these two physiological parameters (Figure 6). For the WN and NP ecotypes, *iWUE*_s_ initially decreased and subsequently increased with increasing *gₛ*, although the magnitude of variation differed between the two ecotypes. In contrast, *iWUE* in the KH ecotype increased rapidly at the beginning and then gradually as *gₛ* increased further. By comparison, *iWUE* in the LA ecotype exhibited a continuous increase with increasing *gₛ*.

### 2.5. Responses of iWUE and gₛ to Change of Water Stress Under Saturating Light

**Figure 7:**
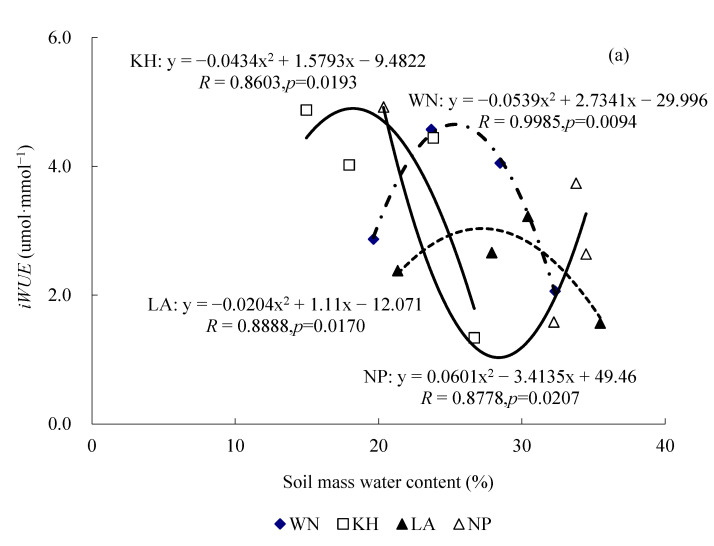

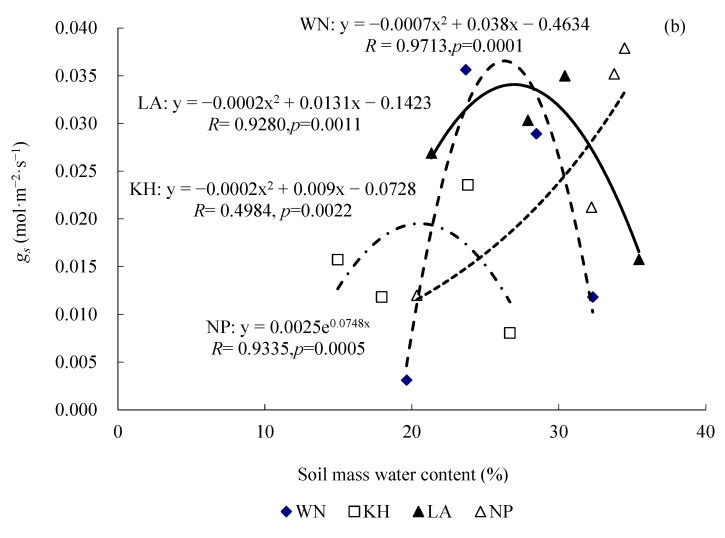
Relationship between the soil mass water content and the instantaneous water use efficiency (*iWUE*) (**a**) and stomatal conductance (*gₛ*) (**b**) measured in seedlings of the four ecotypes of *I. lanceolatum* under saturation light intensity of more than 1500 µmol·m^−2^·s^−1^. (**a**) *iWUE*. (**b**) *gₛ*.

The results indicate pronounced ecotypic differences in the responses of *iWUE* to the light changes in water status under short-term drought stress and subsequent rewatering. Under saturating light conditions (>1500 umol·m^−2^·s^−1^), significant polynomial relationships were observed between *W*_m_ and *iWUE* in seedlings of all four *Illicium lanceolatum* ecotypes (Figure 7a). Specifically, *iWUE* in the KH, LA, and WN ecotypes initially increased and then decreased, along with the increase in *W*_m_, whereas the NP ecotype exhibited the opposite trend, showing an initial decrease followed by an increase (Figure 7a). The polynomial relationships were significantly significant (*R* was 0.8603, 0.8888, 0.9985, and 0.8778 for KH, LA, WN, and NP, respectively), suggesting that *iWUE* is highly sensitive to changes in soil water status under high light conditions. Overall, these results suggest that an optimal water supply under saturating light enhances *iWUE* in the KH, LA, and WN ecotypes, while excessive water availability reduces water use efficiency.

Under the saturating light conditions, significant polynomial function correlations were also detected between *W*_m_ and *gₛ* for the WN, LA, and KH ecotypes (Figure 7b); *R* ranged from 0.4984 to 0.9713, *p* < 0.01. In contrast, the correlation between *W*_m_ and *gₛ* for the NP ecotype belongs to a significant exponential function (*R* = 0.9335, *p* = 0.0005). These findings suggest that *gₛ* in all four ecotypes is more sensitive to *W*_m_ under the saturating light intensity (>1500 µmol·m^−2^·s^−1^).

## 3. Discussion

### 3.1. Responses of I. lanceolatum Photosynthetic Parameters to Drought Stress

Climate change is expected to reduce annual precipitation and prolong dry summers, thereby posing increasing threats to forest ecosystems [14]. Drought stress primarily constrains photosynthesis by inducing stomatal closure and, under severe conditions, by causing damage to palisade mesophyll cells [37,44,45,46,47,48]. Plants with a low LCP and a high LSP generally exhibit greater adaptability to fluctuating light environments; therefore, selecting genotypes capable of tolerating a wide range of light conditions is critical for plant survival under extreme climatic scenarios [45,49]. In the present study, the four *Illicium lanceolatum* ecotypes exhibited distinct strategies of photosynthetic adjustment in response to progressive drought stress. The LSP of the WN ecotype increased initially and then decreased with advancing drought, reaching a maximum value significantly higher than that under the initial water supply after 4 days of drought. In contrast, the LSP of the KH, LA, and NP ecotypes significantly increased after 2 days of drought and subsequently declined. These patterns indicate ecotype-specific drought adaptation strategies mediated through dynamic regulation of LSP. Notably, the WN ecotype maintained a relatively high LSP for a longer drought duration, suggesting an enhanced ability to utilize high light under water-limited conditions.

Previous studies have reported that drought can reduce photosynthesis by 29.5% to 57.7% for *Tilia amurensis* Rupr. seedlings [5]. In this study, the timing and magnitude of the decline in *P*_max_ differed markedly among ecotypes. The NP ecotype experienced the earliest reduction in *P*_max_, occurring after only 2 days of drought, while the WN ecotype had the latest decrease, which occurred after 7 days of drought and remained higher than the initial well-watered level. These contrasting responses likely reflect differences in stomatal regulation and hydraulic behavior among ecotypes, although the underlying mechanisms of stomatal dynamics require further investigation.

Consistent with Li et al. (2019) [44], our study reveals a progressive sequence of leaf photophysiological adjustments in response to increasing drought severity (Figure 1, Figure 2 and Figure 4). Clear ecotypic differences were observed in photosynthetic traits throughout the drought treatment. For the WN ecotype, both *P*_max_ and the LSP peaked when *W*_m_ dropped to 23.67%, after which they declined (Figure 1, Table 1). In contrast, the threshold *W*_m_ values corresponding to declines in *P*_max_ and LSP were 23.81% and 30.41% for the KH and LA ecotypes, respectively (Figure 1, Table 1). These findings indicate that the photosynthetic activities in *I. lanceolatum* seedlings is suppressed when soil moisture drops below an ecotype-specific threshold. Such thresholds provide valuable reference points for optimizing irrigation regimes in artificial cultivation and for defining drought-tolerance limits.

Following rewatering, *P*_max_ increased markedly in the WN ecotype, whereas it continued to decline or recovered slowly in the LA and KH ecotypes. These results suggest that the LA ecotype is more sensitive to drought and high light stress and exhibits limited recovery capacity, whereas the WN ecotype demonstrates both reduced photosynthetic inhibition during drought and strong resilience upon rehydration. Previous metabolomic studies have shown that drought induces the accumulation of amino acids and sugars, implicating these pathways in stress tolerance [50]. The superior post-drought recovery of the WN ecotype observed here may, therefore, be associated with specific metabolic adjustments, a hypothesis that warrants further investigation.

Climatic data from the native habitats of the four ecotypes (Table 2) reveal that the WN ecotype originates from regions with significantly lower annual precipitation than those of the other ecotypes (*p* < 0.05). This long-term exposure to water-limited environments likely underlies its enhanced drought tolerance and recovery capacity. In contrast, although the annual mean temperatures of the WN, KH, and LA habitats are comparable, the NP ecotype originates from a significantly warmer region, which may explain its relatively slower recovery following drought stress.

Rewatering restored the LSP and LCP in all ecotypes, with LSP values in the WN, KH, and LA ecotypes exceeding those under initial water supply conditions. Moreover, AQY increased significantly after drought stress in all ecotypes except NP (*p* < 0.05), indicating that short-term drought enhanced low-light utilization efficiency. These results provide important guidance for optimizing light conditions in *I. lanceolatum* cultivation.

The ecotypic differences observed in this study are consistent with previous reports in other woody species, such as olive (*Olea europaea* L.) [45] and European beech (*Fagus sylvatica* L.) [51], which have demonstrated contrasting drought sensitivity and recovery among genotypes. Collectively, our findings suggest that genetic variation plays a critical role in determining drought adaptive potential, even among ecotypes originating from adjacent subtropical regions [51,52].

### 3.2. Responses of Stomatal Conductance and Water Use Efficiency Response to Light Under Drought Stress

Stomata regulate gas exchange between plants and the atmosphere and are central to plant water and carbon balance [8]. Under drought conditions, reductions in stomatal conductance are often linked to declining hydraulic capacity [8], making stomatal regulation a key determinant of water use efficiency (WUE) and drought survival [20,22]. Understanding these responses is crucial for identifying the type of stomata that optimizes water use efficiency and photosynthetic performance under different environmental conditions [53]. However, stomatal responses to drought vary widely among species, functional types, and genotypes [54,55,56], and remain poorly understood in *I. lanceolatum*.

Previous studies have shown that stomatal conductance declines rapidly with drought in some species while remaining relatively stable in others [54]. Herbaceous plants generally exhibit steeper declines in *g_s_* than woody species, with shrubs and lianas showing intermediate responses [54]. In woody plants, drought strategies range from stomatal closure to maintain hydraulic safety to sustained gas exchange under moderate water stress [22,55,56,57]. Integrating an empirical response into the *g_s_* model based on experimental measurements was a very simple way to simulate the *g_s_* response to drought [58,59].

In the present study, *g_s_* responses to drought differed markedly among *I. lanceolatum* ecotypes. Stomatal conductance decreased under drought in the NP ecotype, consistent with conservative water use strategies reported in other species. In contrast, *g_s_* increased in the WN, KH, and LA ecotypes during short-term drought and remained higher after rewatering than at the onset of the experiment. These contrasting patterns highlight pronounced ecotypic variation in stomatal regulation.

After rewatering, *g_s_* in the WN and KH ecotypes exceeded values observed during drought, whereas *g_s_* in the LA and NP ecotypes remained significantly lower than initial levels. These results suggest that drought-tolerant ecotypes maintain greater stomatal plasticity and recovery capacity. Interestingly, the initial low *g_s_* values observed under well-watered conditions may have resulted from excessive irrigation, potentially causing transient root hypoxia. This finding underscores the importance of avoiding overwatering in cultivation practices for *I. lanceolatum*.

Under saturating light conditions (PAR > 1500 µmol·m^−^^2^·s^−^^1^), significant polynomial relationships were observed between soil water content and *g_s_* in the WN, KH, and LA ecotypes, while the NP ecotype exhibited an exponential relationship. These nonlinear responses differ from the linear relationships reported in some previous studies [60], likely reflecting differences in genetic background, environmental context, and temporal scale of measurement.

Stomatal conductance plays a dual role in regulating photosynthesis and water use efficiency [61,62,63,64]. In this study, dynamic changes in *g_s_* had ecotype-dependent effects on *iWUE*. Under short-term drought and high light conditions, increased *iWUE* was observed across ecotypes, particularly in the WN and KH ecotypes. These results indicate that these two ecotypes are better able to balance carbon gain and water loss under stressful conditions.

Overall, the WN ecotype exhibited superior performance across a wide range of light intensities and water conditions, reflecting broad ecological adaptability. The NP ecotype also showed relatively high tolerance, whereas the KH and LA ecotypes exhibited narrower soil moisture requirements. These findings have practical implications for urban forestry and conservation, where water limitation is increasingly common due to urban warming and climate change [65,66].

Based on our results, we recommend reducing light intensity through shading when soil water availability is limited, as this strategy can mitigate drought-induced stress and enhance *iWUE* in *I. lanceolatum*. This conclusion aligns with previous findings showing improved light use efficiency under shaded conditions in olive trees [45]. Ultimately, successful introduction and cultivation of *I. lanceolatum* across diverse environments should consider both genetic background and environmental interactions, with integrated water and light management strategies tailored to ecotype-specific responses.

## 4. Materials and Methods

### 4.1. Study Site

The experiment was carried out in Tian-mu Mountain Forest Nursery, Lin’an, Zhejiang province, China (118°51′–119°52′ E, 29°56′–30°23′ N). The elevation of the study site was 47 m asl. Central North Asia features a seasonal monsoon climate. The average annual temperature was 15.4 °C and the mean annual rainfall was 1600 mm in 2020. The experimental plot was set up on a flat land in front of a slope. The physical and chemical properties of the soil were determined synchronously in this study site before our experiment. Therefore, soils were acidic yellow loam with a pH value of 4.62. The chemical properties of the soils featured total organic matter content of 3.95 g·kg^−1^, total nitrogen of 0.44 g·kg^−1^, total phosphorus of 10.09 g·kg^−1^, total potassium of 6.31 g·kg^−1^, available nitrogen of 217.62 mg·kg^−1^, available phosphorus of 363.97 mg·kg^−1^, and available potassium of 40.51 mg·kg^−1^. Specific determination standards and test methods referred to forestry industry standards of the People’s Republic of China (LY/T 1228-2015, LY/T 1232-2015, LY/T 1234-2015, LY/T 1237-2015, and LY/T 1239-2015) [67,68,69,70,71], and all of the standards drafted by the Research Institute of Forestry, Chinese Academy of Forestry, and issued by the State Forestry and Grassland Administration. Among them, the determination of soil total nitrogen and available nitrogen is based on the standard of LY/T 1228-2015 [67], the determination of soil total phosphorus and available phosphorus is based on the standard of LY/T 1232-2015 [68], the determination of soil total potassium and available potassium is based on the standard of LY/T 1234-2015 [69], the determinations of soil featured total organic matter content is based on the standard of LY/T 1237-2015 [70], and the determination of pH value is based on the standard of LY/T 1239-2015 [71].

### 4.2. Method

#### 4.2.1. Preparation of Experimental Plants

This study focused on four ecotypes from Lin’an Zhejiang (LA, 119°24′–119°28′ E, 30°18′–30°24′ N), Kai’hua Zhejiang (KH, 118°01′–118°37′ E, 28°54′–29°30′ N), Wu’ning Jiangxi (WN, 114°29′–114°36′ E, 28°53′–29°14′ N), and Nan’ping Fujian (NP, 117°10′–117°24′ E, 26°15′–27°19′ N), respectively. Seedlings were raised in an original location for each ecotype. Fruits were harvested from healthy wild plants in each location during September–October in 2020. Air-dried seeds were stored in moist sands at 4 °C. In mid-April 2021, seeds were sown in the field on a 10 m × 1.0 m seed bed with inter-row spacing of 10 cm and in-row spacing of 5 cm.

**Figure 8:**
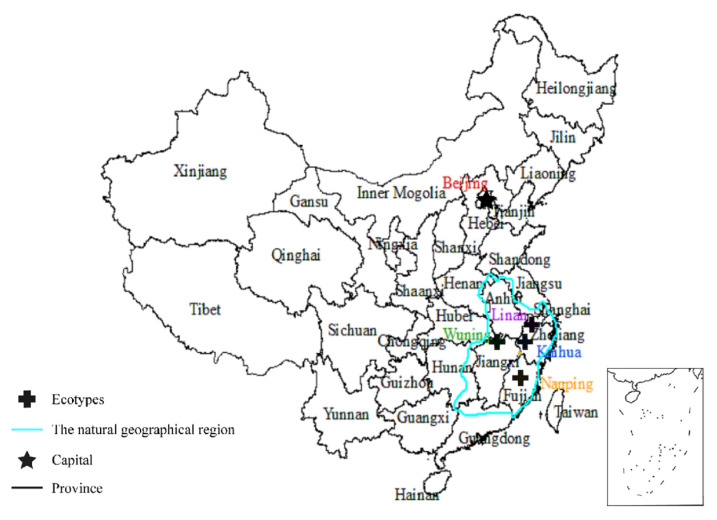
The natural geographical region of this study is marked with the green line. The locations of the four ecotypes of *I. lanceolatum* are marked as black crosses.

The growth medium was a mixture of garden soils and an organic fertilizer (2:1). During the seedling emergence period in early June 2021, the average planting density was 189 plants·m^−2^. This was the seeding emergence density. Later, as the seedlings grew, thinning was carried out to reduce the seedling density avoid shading. Following a routine practice, the field was mulched with straw immediately after seeding, which could provide 50% of shade during the hot summer season, which ranged from July to September in 2021. Irrigation was applied at the seed emergence stage, and seedlings were thinned to the same density at all four locations. One-year-old seedlings were then transferred to the location where this study was conducted. The natural geographical distribution regions and the four ecotypes in this experiment of *I. lanceolatum* are shown in Figure 8, and the climatic conditions of the four original distribution regions for the four ecotypes are provided in Table 2. There was a significant difference among the average annual rainfall for the four ecotypes in their natural growth environments. The annual average temperature of the natural growth environment of the NP ecotype was significantly different from those of the other three ecotypes (Table 2). The growth characters of 50 one-year-old seedlings for each *I. lanceolatum* ecotype are shown in Table 3.

#### 4.2.2. Experimental Design

On 10 June 2022, 10 uniform plants of one-year-old seedlings were selected for each ecotype, and repotted into plastic pots 20 cm wide and 25 cm deep. To prevent the different root systems of the four ecotypes having varying effects on water absorption and utilization intensity, a single-plant-per-pot planting experimental method was adopted. Each pot was filled with 10 kg of a potting mix of sieved yellow soils (2 parts), sands soils (1 part), and humus (1 part). Soil bulk density (*d*) was measured, and then seedlings were transplanted into the pots. One-third of each pot was buried in the ground, and plants were watered regularly. In order to prevent the effect of rainfall on the water stress test, the experiment was conducted in the plastic awning, and the seedlings were given normal illumination in sunny days. All photosynthetic parameters were measured outside the shed.

The fast-growing season begins in mid-July. During the experiment, a soil water probe (ThetaProbe, ML2x, Delta-T Devices, Cambridge, UK) was used to monitor the soil volumetric water content (*W*_v_). For each drought treatment, three replicates of measurements for *W*_v_ were taken, and the mean value was finally calculated and recorded. The soil mass water content (*W*_m_) was estimated using the formula *W*_m_ (%) = *W*_v_ × 100%/d, where *W*_m_ and *W*_v_ are soil mass water content and soil volumetric water content, respectively, and *d* is the mean value of soil bulk density from all the pots. *W*_m_ at the four original seedling growing locations and during different periods of drought treatment is given in Table 1.

Pots were watered weekly. On 20 July 2022, after sufficient watering until it reached a constant weight, drought treatment was initiated, photosynthesis was measured on the same day, and data of pre-drought treatment was recorded. Subsequent measurements were taken at 2 d, 4 d, and 7 d after last well watering and during the recovery stage of rewatering. The measurement was performed on the second day after rewatering sufficiently until it reached a constant weight.

#### 4.2.3. Photosynthesis and Changes in Light Response Parameters

Photosynthesis was measured using a portable photosynthesis system (Licor-6400, Li-Cor Inc., Lincoln, NE, USA) on the day of watering (control); 2 d, 4 d, 7 d after drought treatments; and during the recovery period after watering was resumed. Five plants from each ecotype were selected for fixed photosynthesis measurement, and two or three of the upper south facing canopy leaves were used randomly for the measurements with one reading of each leaf. During measurement of light responses of photosynthetic parameters, the red–blue light source was provided by the Licor-6400. Measurements were conducted at an air flow rate of 0.5 L·min^−1^, leaf temperature of 26 °C, relative humidity of 60%, and CO_2_ concentration of 400 µmol·mol^−1^. Each measurement followed an illumination gradient and started at 2000 µmol·m^−2^·s^−1^ of PAR, and gradually decreased to 1800, 1500, 1200, 1000, 800, 500, 200, 120, 80, 50, 20, and 0 µmol·m^−2^·s^−1^.

A light response curve was generated using data taken on sunny days from 9:00–11:00 a.m. A net photosynthetic rate (*P*_n_)–photosynthetic active radiation (*PAR*) curve was fitted to a nonrectangle hyperbola model [72] to estimate photosynthetic parameter values [72]. The net photosynthetic rate (*P*_n_), stomatal conductance (*gₛ*), and the transpiration rate (E) were determined using the Licor-6400 portable photosynthesis system (Licor-6400, Li-Cor Inc., Lincoln, NE, USA). At the leaf level, instantaneous water use efficiency (*iWUE*) was calculated by dividing the net photosynthetic rate by the transpiration rate (*A*/E) [36].

#### 4.2.4. Data Analysis

Microsoft Excel 2003 (11.0, Microsoft Corporation, Redmond, WA, USA) and SPSS16.0 (SPSS Corporation, Chicago, IL, USA) were used to conduct the statistical analyses. Two-way analysis of variance (ANOVA) (*p* < 0.05) was used in the statistical software SPSS16.0 to compare the effect of the drought stress and duration time on the photosynthetic parameters according to different ecotypes. In this study, two-way analysis of variance showed that the differences among different drought treatments, among different ecotypes, and the interaction between drought and ecotype were all significant (*p* < 0.01). Subsequently, the least significant difference method (LSD) was used to perform post hoc comparisons to examine the significant differences of various photosynthetic physiological parameters among different drought treatments and among different provenances. The correlation between the *gₛ* and the *iWUE* was also analyzed for each ecotype according to different lengths in the drought period.

## 5. Conclusions

Under global climate change, drought has become an increasingly serious constraint on plant growth and productivity. This study demonstrates clear ecotypic differences in photosynthetic performance, *gₛ*, and *iWUE* in four ecotypes of *Illicium lanceolatum* in response to short-term drought stress.

Compared with well-watered conditions, photosynthetic capacity was moderately enhanced after 2–4 days without watering, accompanied by increased *gₛ* across ecotypes. This response likely reflects alleviation of root hypoxia caused by excessive watering prior to drought onset. Under saturating light intensity (>1500 μmol·m^−^^2^·s^−^^1^), significant polynomial relationships were observed between soil water content (*W_m_*) and *gₛ* in the WN, LA, and KH ecotypes, indicating strong stomatal sensitivity to soil moisture under high irradiance.

Following rewatering, *g_s_* in the WN and KH ecotypes exceeded values observed during drought, whereas *g_s_* in the LA and NP ecotypes remained significantly lower than those under initial well-watered conditions. Short-term soil drought combined with high light intensity promoted *iWUE* across ecotypes, with the WN ecotype exhibiting the widest ecological adaptability. This ecotype maintained strong stomatal regulation under severe drought, enabling relatively high photosynthetic potential and efficient use of light and water resources.

Overall, drought stress enhanced leaf sensitivity to high light, and both water limitation and light inhibition were the dominant factors driving photosynthetic decline among ecotypes. These findings highlight the importance of considering interactions between genetic background (ecotype) and environmental factors (water availability and light intensity) when introducing *I. lanceolatum* into new climatic regions. Optimized irrigation and shading strategies should, therefore, be integrated into management practices to achieve high-efficiency cultivation under increasingly water-limited conditions.

## Figures and Tables

**Table 1:** The soil water contents (mean ± standard deviation) of one-year-old seedlings from four *I. lanceolatum* ecotypes across treatment phases. (*n* = 3, *p* < 0.05).

Ecotype	Soil Mass Water Content (%)			
	Initial Stage	Drought for 2 Days	Drought for 4 Days	Drought for 7 Days
WN	32.31 ± 1.45 a	28.47 ± 1.11 bc	23.67 ± 1.32 bc	19.64 ± 0.34 a
KH	26.67 ± 0.23 b	23.81 ± 1.36 b	17.95 ± 0.65 c	14.96 ± 0.09 b
LA	35.45 ± 0.78 a	30.41 ± 0.89 ac	27.89 ± 1.12 b	21.33 ± 0.14 a
NP	34.47 ± 2.01 a	33.77 ± 0.45 a	32.22 ± 2.45 a	20.34 ± 1.02 a
Mean	32.23 ± 3.92	29.12 ± 4.16	25.43 ± 6.10	19.07 ± 2.82

Note: Different lower case letters (a, b, c, etc.) listed after the numbers in each column indicate significant differences among ecotypes under the same treatment according to LSD test (*p* < 0.05).

**Table 2:** Climatic conditions in the native origins of the four *I. lanceolatum* ecotypes.

Ecotype	Abbreviation of Ecotype	North	East	Mean Annual Precipitation (mm)	Elevation Above Sea Level (m)	Mean Annual Sunshine Time (h)	Mean Annual Temperature (°C)	Extreme Maximum Temperature (°C)	Extreme Minimum Temperature (°C)
Wu’ning	WN	28°53′–29°14′	114°29′–114°36′	1400 ± 4.90 d	375 ± 1.44 d	1700 ± 7.07 c	16.2 ± 0.14 b	42.0 ± 0.28 ab	−5.0 ± 0.07 a
Kai’hua	KH	28°54′–29°30′	118°01′–118°37′	1700 ± 4.24 b	437 ± 1.14 a	1775 ± 3.53 b	16.3 ± 0.07 b	41.1 ± 0.01 b	−10.0 ± 0.11 b
Lin’an	LA	30°18′–30°24′	119°24′–119°28′	1650 ± 6.36 c	425 ± 3.50 b	1940 ± 7.97 a	16.1 ± 0.01 b	42.0 ± 0.14 a	−13.8 ± 0.21 c
Nan’ping	NP	26°15′–27°19′	117°10′–117°24′	1800 ± 4.95 a	412 ± 3.56 c	1720 ± 3.41 c	21.1 ± 0.63 a	37.5 ± 0.16 c	−5.5 ± 0.14 a

Note: Lower case letters (a, b, c, d, etc.) following values within the same column indicate significant differences among ecotypes for a given climatic variable, based on the LSD test (*p* < 0.05).

**Table 3:** The growth characteristics (mean ± standard deviation) of one-year-old seedlings for the four different ecotypes of *I. lanceolatum* (*n* = 50, *p* < 0.05).

Ecotypes	Mean Ground Diameter (cm)	Mean Height (cm)	Mean Height Under Branch (cm)	Mean Crown Diameter (cm)	Longest Lateral Branch (cm)
WN	1.05 ± 0.14 a	85.7 ± 1.02 a	34.1 ± 0.13 a	33.3 ± 0.80 a	22.7 ± 0.96 b
KH	0.99 ± 0.15 a	84.2 ± 1.24 a	18.7 ± 0.09 b	30.5 ± 0.66 b	26.7 ± 1.05 a
LA	1.00 ± 0.17 a	81.2 ± 0.98 b	17.2 ± 0.15 c	29.3 ± 0.64 b	21.1 ± 0.94 bc
NP	1.04 ± 0.14 a	83.9 ± 0.80 ab	15.0 ± 0.21 d	28.9 ± 0.70 b	17.8 ± 0.83 c

Note: Different lower case letters (a, b, c, d, etc.) listed after the numbers in each column indicate significant differences among the ecotypes under the same growth characteristic according to LSD test (*p* < 0.05).

## Data Availability

The original contributions presented in this study are included in the article. Further inquiries can be directed to the corresponding author.

## References

[1] Khan Z., Jan R., Asif S., Farooq M., Jang Y.H., Kim E.G., Kim N., Kim K.M. (2024). Exogenous melatonin induces salt and drought stress tolerance in rice by promoting plant growth and defense system. Sci. Rep..

[2] Ali S., Mir R.A., Haque M.A., Danishuddin, Almalki M.A., Alfredan M., Khalifa A., Mahmoudi H., Shahid M., Tyagi A. (2025). Exploring physiological and molecular dynamics of drought stress responses in plants: Challenges and future directions. Front. Plant Sci..

[3] Guan X.Y., Jansen S., Huang L.X., Chen S.L., Zhu S.D. (2025). Evergreen species exhibit higher growth resistance under drought: Insights from carbon-water relations. Tree Physiol..

[4] Niemczyk M., Wrzesiński P., Wojda T., Mohytych V. (2025). High phenotypic variation in growth, stomatal regulation, and water-use efficiency in *Quercus robur* L. provenances may suffice for climate change adaptation. For. Ecol. Manag..

[5] Liu D., Wang Z.G., Han B.X., Mencuccini M., Camarero J.J., Xie Y.S., Zhao B.Q., Wang X.C. (2025). Plasticity of physiological, anatomical and structural traits defines seedling growth during sustained drought. Tree Physiol..

[6] Theroux Rancourt G., Ethier G., Pepin S. (2015). Greater efficiency of water use in poplar clones having a delayed response of mesophyll conductance to drought. Tree Physiol..

[7] Allen B.S., Stewart J.J., Polutchko S.K., Ocheltree T.W., Gleason S.M. (2025). Long-term in vivo observation of Maize leaf xylem embolism, transpiration and photosynthesis during drought and recovery. Plant Cell Environ..

[8] Martin-StPaul N., Delzon S., Cochard H. (2017). Plant resistance to drought depends on timely stomatal closure. Ecol. Lett..

[9] Chaplin E., Merchant A., Salter W. (2025). Smarter stomata: Emergent technologies unlocking yield potential in a changing climate. AoB Plants.

[10] Sperry J.S., Love D.M. (2015). What plant hydraulics can tell us about responses to climate-change droughts. New Phytol..

[11] Tarin T., Eamus D., Santini N.S., Nolan R.H. (2024). Contrasting regulation of leaf gas exchange of semi-arid tree species under repeated drought. Tree Physiol..

[12] Brodribb T.J., Holbrook N.M., Edwards E.J., Gutierrez M.V. (2003). Relations between stomatal closure, leaf turgor and xylem vulnerability in eight tropical dry forest trees. Plant Cell Environ..

[13] Keenan T.F., Hollinger D.Y., Bohrer G., Dragoni D., William J., Schmid H.P., Richardson A.D. (2013). Increase in forest water-use efficiency as atmospheric carbon dioxide concentrations rise. Nature.

[14] Laoué J., Gea-Izquierdo G., Dupouyet S., Conde M., Fernandez C., Ormeño E. (2024). Leaf morpho-anatomical adjustments in a *Quercus pubescens* forest after 10 years of partial rain exclusion in the field. Tree Physiol..

[15] Ramya K.T., Bellundagi A., Harikrishna, Rai N., Jain N., Singh P.K., Arora A., Singh G.P., Prabhu K.V. (2021). Gene action governing the inheritance of stomatal conductance in four wheat crosses under high temperature stress condition. Front. Plant Sci..

[16] Wang C., Li Z., Chen Y., Zhu J., Wang J., Zhao Y., Ouyang L., Zhao H. (2024). Characteristic changes in compound drought and heatwave events under climate change. Atmos. Res..

[17] Kannenberg S.A., Guo J.S., Novick K.A., Anderegg W.R., Feng X., Kennedy D., Konings A.G., Martínez-Vilalta J., Matheny A.M. (2022). Opportunities, challenges and pitfalls in characterizing plant water-use strategies. Funct. Ecol..

[18] Medlyn B.E., De Kauwe M.G., Lin Y.S., Knauer J., Duursma R.A., Williams C.A., Arneth A., Clement R., Isaac P., Limousin J.M. (2017). How do leaf and ecosystem measures of water-use efficiency compare?. New Phytol..

[19] Adams M.A., Buckley T.N., Turnbull T.L. (2020). Diminishing CO_2_-driven gains in water-use efficiency of global forests. Nat. Clim. Change.

[20] Davidson K.J., Lamour J., Rogers A., Ely K.S., Li Q.Y., McDowell N.G., Pivovaroff A.L., Wolfe B.T., Joseph Wright S., Zambrano A. (2022). Short-term variation in leaf-level water use efficiency in a tropical forest. New Phytol..

[21] Yang S.S., Zhang J.H., Han J.Q., Yun B., Lan X., Zhang S., Cao D., Wang J.W. (2025). The ratio of transpiration to evapotranspiration dominates ecosystem water use efficiency response to drought. Agric. For. Meteorol..

[22] Klein T., Shpringer I., Fikler B., Elbaz G., Cohen S., Yakir D. (2013). Relationships between stomatal regulation, water-use, and water-use efficiency of two coexisting key Mediterranean tree species. For. Ecol. Manag..

[23] Wang X., Chen G., Wu M.Q., Li X.Z., Wu Q., Wang P., Zeng H., Yang R., Tang X.L. (2022). Differences in the patterns and mechanisms of leaf and ecosystem-scale water use efficiencies on the Qinghai-Tibet Plateau. Catena.

[24] Zhu K., Zuo Q.H., Liu F.W., Qin J.M., Wang A.Z., Zhang J., Flexas J. (2024). Divergences in leaf CO_2_ diffusion conductance and water use efficiency of soybean coping with water stress and its interaction with N addition. Environ. Exp. Bot..

[25] Flexas J., Bota J., Loreto F., Cornic G., Sharkey T.D. (2004). Diffusive and metabolic limitations to photosynthesis under drought and salinity in C3 plants. Plant Biol..

[26] Medrano H., Tomas M., Martorell S., Flexas J., Hernández E., Rosselló J., Pou A., Escalona J.M., Bota J. (2015). From leaf to whole-plant water use efficiency (WUE) in complex canopies: Limitations of leaf WUE as a selection target. Crop J..

[27] Baby J., Minimol J.S., Santhoshkumar A.V., Joseph J.J., Abd-EIGawad A.M., Ullah F. (2025). Identification and development of drought-tolerant cocoa hybrids: Physiological insights for enhanced water use efficiency under water stress conditions. BMC Plant Biol..

[28] Beikircher B., Mayr S. (2009). Intraspecific differences in drought tolerance and acclimation in hydraulics of *Ligustrum vulgare* and *Viburnum lantana*. Tree Physiol..

[29] Goessen R., Isabel N., Wehenkel C., Gonzales-Vigil E., Hui O., Touchette L., Gagné J., Lamara M., Bousquet J., Mock K.E. (2025). Characterizing genetic adaptations and plastic stress responses within a transcontinental North American keystone species. Tree Physiol..

[30] Aubin I., Munson A.D., Cardou F., Burton P., Isabel N., Pedlar J., Paquette A., Taylor A., Delagrange S., Kebli H. (2016). Traits to stay, traits to move: A review of functional traits to assess sensitivity and adaptive capacity of temperate and boreal trees to climate change. Environ. Rev..

[31] Alzahrani I.J., Magos Brehm J., Maxted N. (2025). Gap Analysis of priority medicinal plant species in the Kingdom of Saudi Arabia. Plants.

[32] Lin Q. (2002). Medicinal plant resources of *Illicium* L.. Chin. Tradit. Herb. Drugs.

[33] Zhao C., Ling H.E., Zhang L.Y., Shan M. (2009). Anti-inflammatory and analgesic effects of two extracts isolated from *Illicium*. Chin. J. Nat. Med..

[34] Abrams M.D., Kloeppel B.D., Kubiske M.E. (1992). Ecophysiological and morphological responses to shade and drought in two contrasting ecotypes of *Prunus serotina*. Tree Physiol..

[35] Seibt U., Rajabi A., Griffiths H., Berry J.A. (2008). Carbon isotopes and water use efficiency: Sense and sensitivity. Oecologia.

[36] Gilbert M.E., Zwieniecki M.A., Holbrook N.M. (2011). Independent variation in photosynthetic capacity and stomatal conductance leads to differences in intrinsic water use efficiency in 11 soybean genotypes before and during mild drought. J. Exp. Bot..

[37] Sensuła B., Wilczyński S. (2022). Dynamics changes in basal area increment, carbon isotopes composition and water use efficiency in pine as response to water and heat stress in Silesia, Poland. Plants.

[38] Pflug E.E., Buchmann N., Siegwolf R.T.W., Schaub M., Rigling A., Arend M. (2018). Resilient leaf physiological response of european beech (*Fagus sylvatica* L.) to summer drought and drought release. Front. Plant Sci..

[39] Silim S., Nash R., Reynard D., White B., Schroeder W. (2009). Leaf gas exchange and water potential responses to drought in nine poplar (*Populus* spp.) clones with contrasting drought tolerance. Trees.

[40] Zhou H.H., Chen Y.N., Li W.H., Chen Y.P. (2010). Photosynthesis of *Populus euphratica* in relation to groundwater depths and high temperature in arid environment, northwest China. Photosynthetica.

[41] Tartachnyk I.I., Blanke M.M. (2004). Effect of delayed fruit harvest on photosynthesis, transpiration and nutrient remobilization of apple leaves. New Phytol..

[42] Nunes C., Araújo S.S., Silva J.M., Fevereiro P., Silva A.B. (2010). Photosynthesis light curves: A method for screening water deficit resistance in the model legume *Medicago truncatula*. Ann. Appl. Biol..

[43] Fan B.L., Ma Z.Q., Gao P.F., Lu J., Ding N., Sun K. (2022). Functional traits of male and female leaves of *Hippophae tibetana* on the eastern edge of the Tibetan Plateau and their altitudinal variability. Plants.

[44] Li Y.L., Liu X.G., Hao K., Yang Q.L., Yang X.Q., Zhang W.H., Cong Y. (2019). Light-response curve of photosynthesis and model fitting in leaves of *Mangifera indica* under different soil water conditions. Photosynthetica.

[45] Sofo A., Dichio B., Montanaro G., Xiloyannis C. (2009). Photosynthetic performance and light response of two olive cultivars under different water and light regimes. Photosynthetica.

[46] Ruzana Adibah M.S., Ainuddin A.N. (2011). Epiphytic plants responses to light and water stress. Asian J. Plant Sci..

[47] Schuldt B., Buras A., Arend M., Vitasse Y., Beierkuhnlein C., Damm A., Gharun M., Grams T.E.E., Hauck M., Hajek P. (2020). A first assessment of the impact of the extreme 2018 summer drought on Central European forests. Basic Appl. Ecol..

[48] Cocozza C., de Miguel M., Pšidová E., Ditmarová L., Marino S., Maiuro L., Alvino A., Czajkowski T., Bolte A., Tognetti R. (2016). Variation in ecophysiological traits and drought tolerance of beech (*Fagus sylvatica* L.) seedlings from different populations. Front. Plant Sci..

[49] Montanaro G., Dichio B., Xiloyannis C. (2009). Shade mitigates photoinhibition and enhances water use efficiency in kiwifruit under drought. Photosynthetica.

[50] Tong R., Zhou B.Z., Cao Y.H., Ge X.G., Jiang L.N. (2020). Metabolic profiles of moso bamboo in response to drought stress in a field investigation. Sci. Total Environ..

[51] Netzer F., Thöm C., Celepirovic N., Ivankovic M., Alfarraj S., Dounavi A., Simon J., Herschbach C., Rennenberg H. (2016). Drought effects on C, N and P nutrition and the antioxidative system of beech (*Fagus sylvatica* L.) seedlings depend on geographic origin. J. Plant Nutr. Soil Sci..

[52] Bolte A., Czajkowski T., Cocozza C., Tognetti R., de Miguel M., Pšidová E., Ditmarová Ĺ., Dinca L., Delzon S., Cochard H. (2016). Desiccation and mortality dynamics in seedlings of different european beech (*Fagus sylvatica* L.) populations under extreme drought conditions. Front. Plant Sci..

[53] Burgess A.J., Ugalde J.M. (2024). SOS: Speed of stomata opening and closing is influenced by vapor pressure deficit. Plant Physiol..

[54] Zhou S.X., Duursma R.A., Medlyn B.E., Kelly J.W.G., Prentice I.C. (2013). How should we model plant responses to drought? An analysis of stomatal and non-stomatal responses to water stress. Agric. For. Meteorol..

[55] Ma W.T., Tcherkez G., Wang X.M., Schäufele R., Schnyder H., Yang Y.S., Gong X.Y. (2020). Accounting for mesophyll conductance substantially improves ^13^C-based estimates of intrinsic water-use efficiency. New Phytol..

[56] Farquhar G.D., von Caemmerer S., Berry J.A. (1980). A biochemical model of photosynthetic CO_2_ assimilation in leaves of C3 species. Planta.

[57] Aranda I., Cano F.J., Gascó A., Cochard H., Nardini A., Mancha J.A., López R., Sánchez-Gómez D. (2015). Variation in photosynthetic performance and hydraulic architecture across European beech (*Fagus sylvatica* L.) populations supports the case for local adaptation to water stress. Tree Physiol..

[58] Damour G., Simonneau T., Cochard H., Urban L. (2010). An overview of models of stomatal conductance at the leaf level. Plant Cell Environ..

[59] Ngao J., Adam B., Saudreau M. (2017). Intra-crown spatial variability of leaf temperature and stomatal conductance enhanced by drought in apple tree as assessed by the RATP model. Agric. For. Meteorol..

[60] El-Sharkawy M.A. (2004). Cassava biology and physiology. Plant Mol. Boil..

[61] Drake P.L., Froend R.H., Franks P.J. (2013). Smaller, faster stomata: Scaling of stomatal size, rate of response, and stomatal conductance. J. Exp. Bot..

[62] Deans R.M., Brodribb T.J., Busch F.A., Farquhar G.D. (2019). Plant water-use strategy mediates stomatal effects on the light induction of photosynthesis. New Phytol..

[63] Eyland D., Wesemael J.V., Lawson T., Carpentier S. (2021). The impact of slow stomatal kinetics on photosynthesis and water use efficiency under fluctuating light. Plant Physiol..

[64] Ozeki K., Miyazawa Y., Sugiura D. (2022). Rapid stomatal closure contributes to higher water use efficiency in major C4 compared to C3 *Poaceae* crops. Plant Physiol..

[65] Horike H., Kinoshita T., Kume A., Hanba Y.T. (2021). Responses of leaf photosynthetic traits, water use efficiency, and water relations in five urban tree species under drought stress and recovery. Trees.

[66] Yadav M., Gupta P., Seth C.S. (2022). Foliar application of α-lipoic acid attenuates cadmium toxicity on photosynthetic pigments and nitrogen metabolism in *Solanum lycopersicum* L.. Acta Physiol. Plant..

[67] (2016). Determination of Forest Soil Nitrogen.

[68] (2016). Phosphorus Determination Methods of Forest Soils.

[69] (2016). Potassium Determination Methods of Forest Soils.

[70] (2016). Organic Matter Determination Methods of Forest Soils.

[71] (2016). pH Determination Methods of Forest Soils.

[72] Thornley J.H.M. (1976). Mathematical Models in Plant Physiology.

